# Efficacy of Tuberculosis Treatment in Patients with Drug-Resistant Tuberculosis with the Use of Bedaquiline: The Experience of the Russian Federation

**DOI:** 10.3390/antibiotics11111622

**Published:** 2022-11-14

**Authors:** Anna Starshinova, Irina Dovgalyk, Ekaterina Belyaeva, Anzhela Glushkova, Nikolay Osipov, Dmitry Kudlay

**Affiliations:** 1 Almazov National Medical Research Centre, 197341 Saint-Petersburg, Russia; 2 St. Petersburg Research Institute of Phthisiopulmonology, 191036 Saint-Petersburg, Russia; 3 Republic TB Healthcare Dispensary, 185032 Petrozavodsk, Russia; 4 V.M. Bekhterev National Research Medical Center for Psychiatry and Neurology, 192019 Saint-Petersburg, Russia; 5 St. Petersburg Department of Steklov Mathematical Institute of Russian Academy of Sciences, 191023 Saint-Petersburg, Russia; 6 St. Petersburg State University, 199034 Saint-Petersburg, Russia; 7 Federal State Autonomous Educational Institution of Higher Education “First Moscow State Medical University Named after I.I. THEM. Sechenov” of the Ministry of Health of Russia (Sechenov University), St. Trubetskaya 8, Building 2, 119048 Moscow, Russia; 8 SSC Immunology Institute, 115552 Moscow, Russia

**Keywords:** tuberculosis, bedaquiline, treatment efficacy, multidrug resistance, extensive drug resistance, mycobacterium tuberculosis

## Abstract

In the conditions of the continued growth of multiple- and extensive drug-resistant tuberculosis, use of the new highly effective anti-tuberculosis drugs in this patient category is of great relevance. The aim of the study was determination the efficacy of treatment in patients with multidrug- and extensive drug-resistant tuberculosis using bedaquiline based on studies published in the Russian Federation. Materials and methods: The authors analyzed data published in studies from 2014 to 2022; 41 publications were included in total and 17 articles corresponded to the study design. The results of treatment of 1404 tuberculosis patients with MDR/XDR TB were described. Bedaquiline was used according to the standard scheme with a description of the treatment results after 24–26 weeks. Treatment efficacy was estimated according to accepted criteria. Results of the study: The analysis showed that the treatment efficacy on conversion was achieved in 79.5% of cases (95% Cl 76.5–82.3), and recovery was observed in 82.0% of cases (95% Cl 78.6–85.1). Departure from the therapy was observed in rare cases (9.8%; 95% Cl 7.9–12.2). Deaths were recorded in 6.5% of cases (95% Cl 4.9–8.3), which were associated with the severe disease and concomitant pathology in 74.3%. The development of adverse events was noted in half of the patients (55.7%); however, bedaquiline cancellation occurred in a few cases (7.0%; 95% Cl 3.0–13.0). From analyzing data in patients with MDR and XDR TB, the efficacy of treatment was 89.9% (95% Cl 85.9–93.2) and 71.9% (95% Cl 66.2–77.1), respectively. Conclusion: Use of bedaquiline in treatment makes it possible to achieve recovery of patients with MDR/XDR TB in 82.0% of cases with patients dropping out of treatment in 9.8%. At the same time, in patients with MDR TB, recovery was achieved in 89.9% of cases, while in patients with XDR TB, 71.9% of cases recovered.

## 1. Introduction

Before the COVID-19 pandemic, many countries around the world had made significant progress in the fight against tuberculosis, as evidenced by a 9% decrease in incidence and a 14% decrease in mortality from it between 2015 and 2019 [1]. At the same time, after the COVID-19 pandemic, mortality from the disease increased to 1.5 million in 2021 (in 2019 1.4 million deaths were from tuberculosis). Additionally, there was a decrease in the number of registered new cases of tuberculosis (TB) of 18%—from 7.1 million in 2019 to 5.8 million in 2020, which will lead to a delay in the elimination of TB by at least 9 years [2].

Of particular concern are countries with a high burden of the disease, including countries with high levels of multidrug-resistant tuberculosis (MDR TB) and HIV infection. This is due to the termination of patient support programs and a decrease in the effectiveness of tuberculosis treatment in the future [3,4].

In the Russian Federation, according to the data of the Russian Research Institute of Health of the Ministry of Health, thanks to the measures taken, the overall incidence of tuberculosis in 2019 decreased by 7.2% (from 44.4 to 41.2 per 100,000 population) compared to 2018. In comparison to 2008 (85.1 per 100,000 population), the decrease was 51.6%. The mortality rate from tuberculosis in 2019 was 5.2 per 100,000 population (in 2018—5.9 per 100,000 population) [5].

A big success in 2019 was the fact that the incidence of multidrug-resistant tuberculosis (MDR TB) of the pathogen to anti-tuberculosis drugs (ATD) among registered patients was not increasing: 4.0 per 100,000 population in 2009; 5.6 per 100,000 population in 2018 and 5.4 per 100,000 population in 2019 [6,7]. However, there was growth of TB patients with MDR TB among patients with pulmonary TB with bacterial excretion, which is a negative prognostic factor [8].

One of the main tasks in the fight against the global problem of tuberculosis is the issue of availability of anti-tuberculosis treatment for patients [1,3]. Of particular relevance is using new effective drugs for patients with multiple and extensive drug resistance of the pathogen [9,10,11]. According to the WHO report, in the period 2018–2019, around 14 million people worldwide have received TB treatment. That it is over one-third of the way to the five-year target (2018–2022) of 40 million people [3].

Bedaquiline is one of the new drugs used in the most severe category of patients; it belongs to the group of diarylquinolines, a new class of anti-tuberculosis compounds [12]. The bactericidal effect of bedaquiline is due to specific inhibition of the proton pump of mycobacteria ATP synthase (adenosine 5’triphosphate synthase), an enzyme that plays a major role in the process of cellular respiration of the *Mycobacterium tuberculosis (Mbt)* [13,14]. Inhibition of ATP synthesis leads to disruption of energy production and, as a result, to the death of microbial cells [3,12,15].

In vitro clinical studies have shown that bedaquiline is active against both drug-susceptible and drug-resistant (including multidrug-resistant, and pre-extensively drug-resistant*) strains of *Mbt* with a minimum inhibitory concentration (MIC) in the range ≤0.008–0.12 µg/mL (MIC 50 0.03 µg/mL and MIC 90 0.06 µg/mL) [16,17,18]. It has been noted that at low concentrations, bedaquiline can exhibit a bacteriostatic effect and potentiate the risk of developing resistance; at high concentrations, it has a bactericidal effect [9,15].

Recent studies have shown the efficacy of bedaquiline not only in adults, but in children over 6 years old. This is part of a combination therapy for pulmonary tuberculosis caused by strains of *Mycobacterium tuberculosis* with multidrug resistance of the pathogen [19,20].

Since 2014, bedaquiline has been involved in practice activities in the Russian Federation, and has been used in adult patients with multidrug and extensively drug-resistant pulmonary tuberculosis, including in combination with HIV infections [21]. Summarizing the experience gained and obtaining new data on the efficacy of the treatment, and the development of adverse events during therapy, is important information for further use of the drug. 

The aim of the study was to determine the efficacy of treatment in patients with multidrug- and extensively drug-resistant tuberculosis using bedaquiline, based on studies published in the Russian Federation.

## 2. Materials and Methods

An analysis was made of the results of studies published from 2014 to 2022 in PubMed, Web of Science, SCOPUS, and Elibrary using the keywords: bedaquiline, tuberculosis, treatment of tuberculosis, drug resistance, multiple and extensive drug resistance pathogen, anti-tuberculosis drugs, the Russian Federation. 

**Figure 1:**
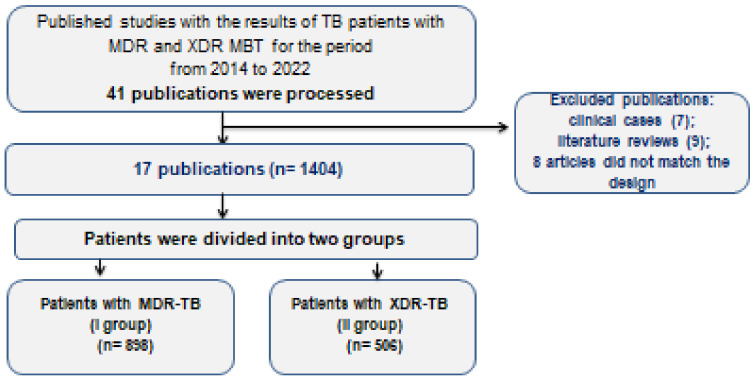
Study design and selection of publications.

There were 112 publications in total; 41 publications were processed, of which 17 articles were included, because they contained the required data, including the necessary data about antimicrobial susceptibility tests. Other publications included: 7 articles describing clinical cases, 9 were reviews of the literature, and 8 articles did not match the study design (Figure 1).

According to the inclusion criteria, studies were included with data presentation of the treatment results of patients with drug-resistant (DR) tuberculosis (TB) (including multidrug-resistant (MDR), and extensively drug-resistant (XDR)) pathogens over the age of 18 years old. 

Studies with a literature review, with data of treatment in patients under 18 years old, data on treatment of pregnant women, clinical case descriptions, and studies missing data for analysis were excluded from our study. 

Data analysis was carried out based on the results of 17 studies, where 1404 patients with drug-resistant tuberculosis were examined and treated. 

All patients underwent a complex bacteriological examination with the determination of the spectrum of drug sensitivity of *Mycobacterium tuberculosis* to anti-TB drugs for further selection of an adequate scheme of anti-TB therapy.

In 61.6% cases (898), patients had multiple DR and in 38.4% (506)—extensive DR of the pathogen, according to bacteriological examination (Table 1).

When analyzing the data obtained, we noted the absence of a comprehensive description of comorbidities among patients in some studies. Unfortunately, long-term results of treatment patients with MDR and XDR were carried out only in five studies [25,26,30,35,36,37]. In other studies, data analysis was carried out until the end of the drug intake.

The general characteristics of TB patients who received a course of therapy with the inclusion of bedaquiline as part of TB treatment are presented in the Table 2.

Infiltrative pulmonary tuberculosis prevailed in 40.3% of patients, fibrous-cavernous TB occurred in one third of patients (30.9%), disseminated pulmonary tuberculosis was diagnosed in 8.3% of cases. Bacterial excretion was observed in most patients (91.4%), which was confirmed by sputum cultures on liquid and solid media. 

In all studies, patients underwent a comprehensive examination, which was recommended by regulatory documents. 

All patients with pulmonary tuberculosis were prescribed a therapy regimen taking into account the data obtained on the drug sensitivity of the pathogen, body weight, comorbidities in accordance with existing local and international recommendations for tuberculosis with multidrug-resistant and extensively drug-resistant pathogen treatments [3,38,39,40,41]. 

Anti-tuberculosis drugs were prescribed based on mycobacteria drug susceptibility data and included a combination from five to eight drugs which are used in the MDR and XDR TB treatment: high dose of isoniazid (H), ethambutol (E), pyrazinamide (Z), kanamycin (Km)/amikacin (Am), and polypeptide (capreomycin—Cm), fluoroquinolones (Fq), protionamide (Pto), ethionamide (Eto), cycloserine (Cs)/terizidone (Trd), aminosalicylic acid (PAS), linezolid (Lzd), amoxicillin and clavulanic acid (Amx/Clv), imipenem (Imp)/cilastatin (Cln), meropenem (Mpm), bedaquiline (Bq), and thioureidoiminomethylpyridinium perchlorate (Tpp).

Patients received bedaquiline according to the existing instructions and recommendations as part of complex therapy at 400 mg one time per day, for 14 days, then 200 mg 3 times per week, for 22 weeks under the supervision of a specialist [1,4]. In the Russian Federation, bedaquiline was registered in 2013 (JSC Pharmstandard, UfaVITA, Russia, LP-00228).

The diagnostic complex for tuberculosis included an assessment of the clinical manifestations of the disease, radiological changes according to the plain chest radiograph, computed tomography (CT) data, and a laboratory complex of sputum studies for the presence of bacterial excretion with the determination of the drug sensitivity spectrum of mycobacteria tuberculosis (inoculation of material on solid and liquid nutrient media in the BACTECMGIT 960) data.

## 3. Criteria for the Efficacy of TB Treatment

Efficacy criteria was determined according to the existing criteria presented in local and international guidelines of the WHO [32,33,34]. 

A comprehensive analysis of patients was carried out taking into account complaints, anamnesis vitae and medical history, the results of objective examination, bacteriological examination (microscopic examination of sputum to detect *Mycobacterium* using fluorescent microscopy, sputum culture (inoculation of material on solid and liquid nutrient media in the BACTECMGIT 960), and radiation complex of examination (plain chest radiograph in two projections and CT-chest). 

Conversion was the main criterion of efficacy according to the data of bacteriological methods and the results of microscopy. The overall efficacy of the course of the therapy included conversion during the last month of treatment, and earlier before the completion of the general course of therapy. Additionally, the absence of symptoms of intoxication and positive radiological dynamics were assessed during the study. The course of therapy was completed after taking 85% of the doses of therapy after 18–24 weeks of treatment [40].

A course of therapy was ineffective if during the therapy bacterioexcretion persisted, determined by one of the above methods after five or more months of treatment. The patient was taken off treatment when he stopped taking anti-tuberculosis drugs for two months at his request, and the absence of the results of a follow-up examination at further stages of treatment. 

A fatal case was the death from tuberculosis during the course of TB therapy. 

The safety assessment of immunotherapy with the analysis of the severity of adverse events (reactions) was carried out using the international five-point scale of “Criteria for assessing adverse events, Version 6.0” (Common Terminology Criteria for Adverse Events (CTCAE)). According to the presented scale, adverse events (AEs) included any clinical manifestations or laboratory studies data during therapy, which might be associated with the use of drugs. The severity of adverse events (AEs) was not assessed, because it was not a task of the study. 

The meta-analysis was carried out in accordance with the PRISMA protocol (http://www.prisma-statement.org, accessed on 8 August 2022) used for this type of study. Statistical analysis of the obtained results was carried out using the Stata 14 program and the Common Open Software Environment R version 4.1.2 [42]. Descriptive statistics methods based on the analysis of absolute and relative frequencies were used for result presentation. Confidence intervals were calculated using the forestplot package exact method [43]. Graphs were built using the forestplot package for R. The program code, which contains all the necessary details, is available at https://github.com/nicknick85/statistics-for-medicine/blob/main/meta.r (accessed on 8 August 2022).

## 4. Results of the Study

**Figure 2:**
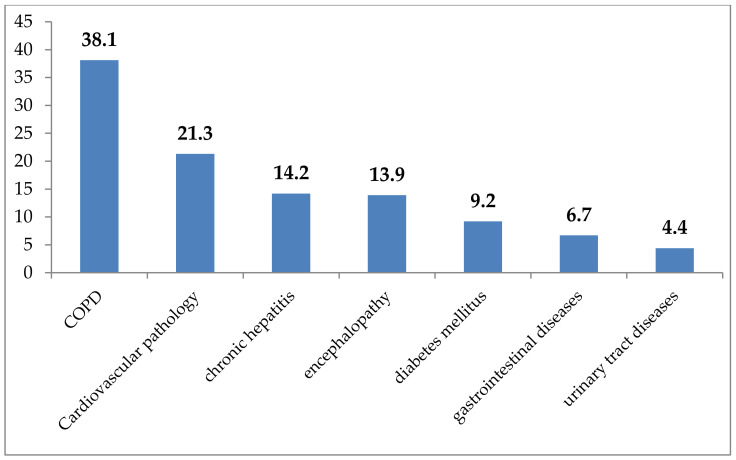
The spectrum of comorbidity in patients with drug-resistant tuberculosis (%).

When analyzing aggravating factors, it was found that the patients were smoking tobacco in half of the cases (58.2%), every fifth had alcohol addiction (20.1%), and in 3.1% of cases, drug addiction was present. In 82.6% of cases, patients had various concomitant diseases, the spectrum of which is shown in the Figure 2.

As shown in Figure 2, 38.1% of patients had chronic obstructive pulmonary disease (COPD), and 21.3% of cases had cardiovascular pathology. Chronic liver pathology and encephalopathy were noted in 14.2% and 13.9% of cases, respectively. In 9.2% of cases, patients suffered from type II diabetes mellitus and diseases of the gastrointestinal tract (6.7%). 

**Figure 3:**
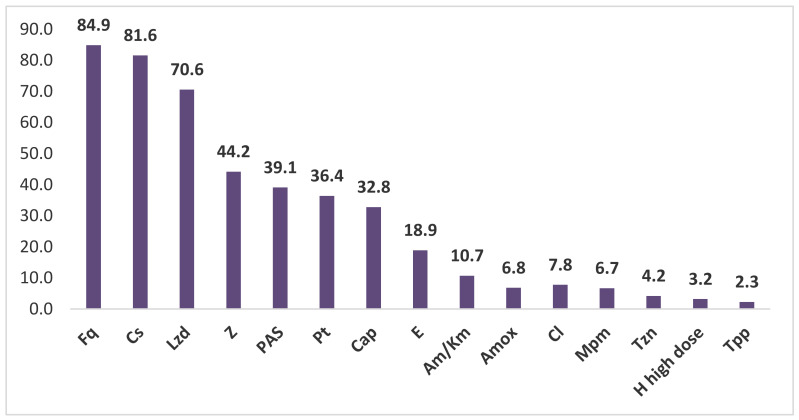
Using drugs in therapy regimens together with bedaquiline (%).

The proportion of each of the anti-tuberculosis drugs included in the bedaquiline therapy regimens is shown in Figure 3.

As shown in Figure 3, Fq, Cs, Lzd, Z, PAS, Pt, and Cap were used more frequently in treatment regimens.

The results of the treatment in patients with MDR and XDR tuberculosis are presented in Table 3. 

**Figure 4:**
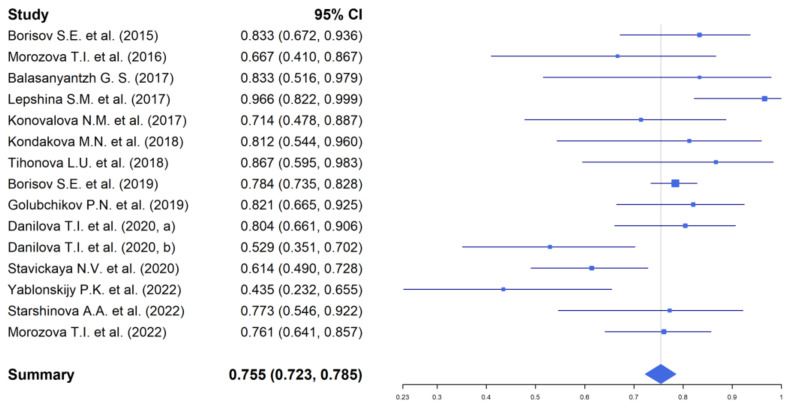
The efficacy of the TB treatment with the use of bedaquiline by the end of 24–26 weeks of therapy. Borisov et al. [22], Morozova et al. [23], Balasanyantzh [24], Lepshina et al. [26], Konovalova et al. [27], Kondakova et al. [28], Tihonova et al. [29], Borisov et al. [30], Golubchikov et al. [31], Danilova et al. [32], Danilova et al. [32], Stavickaya et al. [33], Yablonskiy et al. [35], Starshinova et al. [36], Morozova et al. [37]).

Assessment of the efficacy of therapy after 24–26 weeks of TB treatment (after completion of the course of bedaquiline) was carried out in 576 patients (42.0% of the total number of patients included in the analysis) (Figure 4). 

According to the data, the highest efficacy was obtained by conversion (79.5%). At the same time, recovery was noted in 82.0% of cases. A total of 80 patients were removed from the studies with the discontinuation of therapy.

It should be noted that there were discontinuations of TB treatment in 9.8% of cases and fatal cases due to disease progression in 6.5% of cases.

**Figure 5:**
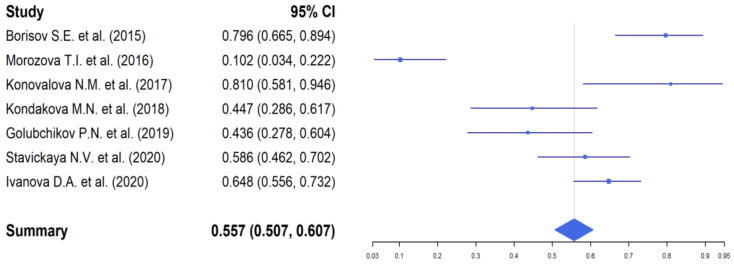
The development of adverse events during therapy with bedaquiline. Borisov et al. [22], Morozova et al. [23], Konovalova et al. [27], Kondakova et al. [28], Golubchikov et al. [31], Stavickaya et al. [33], Ivanova et al. [34].

The development of adverse events (AEs) was noted in half of the cases, 55.7% of the total (95% Cl 50.7–60.7) (Figure 5).

**Figure 6:**
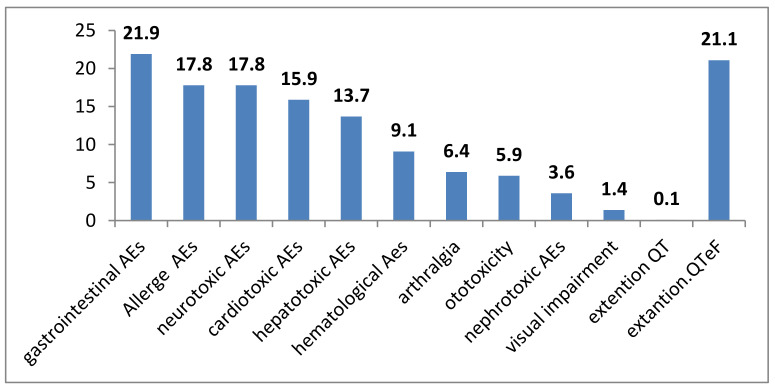
The spectrum of adverse events during therapy with bedaquiline (%). (AEs—adverse events).

The spectrum of adverse events (AEs) in 219 patients with MDR/XDR TB is shown in the Figure 6.

According to the researchers, the most frequent AEs in patients were from the gastrointestinal tract (21.9%), and cardiac arrhythmia with a prolongation of the QTeF interval in 21.1% of cases. Allergic (17.8%) and neurotoxic AEs (17.3%), as well as cardiotoxic (15.9%) and hepatotoxic (13.7%) AEs were noted somewhat less frequently. Violations from other systems were noted in single cases. Cancellation of bedaquiline was required in single cases (7.0%; 95% Cl 3.0–13.0). It was not possible to unequivocally determine the relationship between the development of AEs and bedaquiline use, with the exception of cardiovascular system disorders. 

The overall efficacy of treatment after the end of the course of therapy was evaluated in 388 people and amounted to 82.0% (95% Cl 78.6–85.1). The efficacy of therapy might be different in patients with MDR and XDR TB. We analyzed the data after the end of TB treatment, where bedaquiline was used in the scheme of therapy (Table 4).

In the cohort of patients with MDR tuberculosis, the efficacy of treatment was 89.9%, in the cohort of XDR TB—71.9% 

## 5. Discussion 

According to the WHO, based on reports from 146 countries of the world, only 57% of patients with MDR TB have been successfully treated, in 7% of cases the treatment was ineffective, and in 15% of cases the patients died from the disease [8]. It should be noted that the efficacy of XDR TB treatment did not exceed 34% in the 2018 WHO report [44]. 

In recent years, a large number of preclinical studies have been carried out with the search for drugs that are effective against *M.tuberculosis*, including those with drug resistance, which showed certain results that allow further studies of phase II clinical trials [8,45]. The second phase of clinical trials was conducted to determine the efficacy and safety in TB patients with DR TB when TBA-354, Q203 (imidazopyridine), Sutezolid (PNU-100480—oxazolidinone), OPC-67683 (delamanid), and TMC207 (bedaquiline) are included in the therapy regimen. Trials using diarylquinoline, AZD5847 (oxazolidinone), PBTZ-169 (benzothiazinone derivative), SQ109 (ethylenediamine—analog of ethambutol), tedizolid (a representative of oxazolidones) [9,41], and studies of thioureidoiminomethylpyridinium perchlorate (Tpp) were conducted in the Russian Federation [35,46]. 

According to the results of the studies, only bedaquiline and delamanid were recommended by the WHO for use in the world for the treatment of tuberculosis patients with MDR TB [14]. In the Russian Federation Tpp [38,39] is recommended, which were put into the practice with the possibility of more effective treatment regimens.

Formation of treatment regimens for tuberculosis with the inclusion of bedaquiline made a significant contribution to improving the efficacy of MDR TB and, according to meta-analysis, to achieve successful treatment of up to 61% of patients [47,48]; according to some studies—up to 81% have success [21,22,49]. The combination of drugs is formed by taking into account drug sensitivity of *M.tuberculosis*, which is a key moment in the selection of adequate therapy [33,35,50]. 

In international practice, it was shown that linezolid, fluoroquinolones (levofloxacin or moxifloxacin), carbapenems, clofazimine, etc., were used together with bedaquiline in treatment regimens. This is according to the analysis based on the results of the efficacy of TB treatment using 50 studies from 25 countries of the world [48].

In the Russian Federation, bedaquiline is successfully used in the treatment of patients with MDR and XDR TB, which made it possible to carry out the presented data analysis. In recent years, studies have been conducted with the inclusion of bedaquiline in children of various ages who had a severe and progressive course of MDR TB [51]. 

In general, the results of the research studies in different countries are comparable. The difference lies in the formation of treatment regimens taking into account the available anti-TB drugs, as well as the end of studies after 24–26 weeks of the intensive phase of therapy with bedaquiline. A few researchers were able to get data regarding the efficacy of therapy and long-term results of treatment after the end of the main course of treatment, including a comparison of the results obtained in patients with MDR TB and XDR TB [27,30,36,37]. 

Generalization of the experience gained in the country conditions over a long period of the time is an important point in identifying the advantages of therapy with the inclusion of bedaquiline, and identifying possible disadvantages [52,53]. In the future, identification of existing shortcomings will make it possible to avoid the formation of drug resistance of mycobacteria to new drugs [54,55,56,57], which will limit the possibilities of therapy and reduce its effectiveness.

An important point is the necessity analyzing adverse events against the background of comorbidities in patients [34,58,59,60,61], which have been widely analyzed in multicenter studies with selective inclusion of TB patients from the Russian Federation [21,61]. Obtaining more information about the development of AEs will allow the development of measures to prevent them, in order to increase the efficacy of the patient’s treatment [62,63]. Our study showed that the development of AEs was noted in 54% of cases with gastrointestinal disorders in every fourth patient, and the occurrence of cardiac arrhythmias, but only with a prolongation of the QTeF interval. Registered AEs led to drug withdrawal in 9% of cases, which is consistent with data from international studies [21]. In the international multicenter study [21], which included 428 patients with MDR TB, and from the Russian Federation also, discontinuation of the drug due to the development of adverse events was noted somewhat less frequently, only in 5.6% of cases.

An important difference between the results of treatment and the data available in the literature review is the low percentage of deaths from the disease in patients included in the study. The percentage of deaths did not exceed 6.5%, which is two times lower compared to the data of international studies and the results of a meta-analysis [21,48,52].

## 6. Conclusions

The possibility of including bedaquiline in the regimens for the treatment of DR TB has significantly increased the efficacy of the treatment of this category of patients, especially those with MDR TB. Obviously, TB patients with MDR and XDR TB are the most difficult category of patients with comorbidity in 74% of cases, including 6% of cases occurring with an HIV infection. Despite the existing difficulties, the use of a therapy regimen with the inclusion of bedaquiline for 24–26 weeks using an intensive phase of therapy received data about conversion, as the most significant criterion of efficacy, in 79.5% of cases.

Comparable data were obtained in the meta-analysis, where 29 studies were selected from 2679 articles [47], and treatment efficiency was obtained in 74% of cases. An important component of ongoing therapy is a low percentage of treatment failures (9.8%), which may be due to both increased motivation for the treatment of patients and careful selection of patients for therapy. Obviously, the inclusion of only one new drug in the treatment regimen for patients with XDR TB is not sufficient [52]. It is necessary to include a larger number of new and effective anti-tuberculosis drugs in order to form a treatment regimen for at least four of them, which will further reduce the duration of therapy while maintaining the achieved results, including long-term ones [9,53].

Since 2022 in the Russian Federation, it is necessary to include delamanid, which has been recommended by the WHO since 2015, in the scheme of therapy for MDR TB patients. Experience with the combined use of bedaquiline and delamanid is accumulating [47], but predictively, one can speak of a higher treatment efficacy compared to the inclusion of bedaquiline alone in the therapy regimen with no increase in adverse events.

## Figures and Tables

**Table 1:** Data on publications and enrolled patients.

N	Author Surname, First Name, Patronymic	Year	Number of Patients	Follow-Up Period (Weeks)	MDR MTB (n)	%	XDR MTB (n)	%
1	Borisov S.E. et al. [22]	2015	54	24	23	42.6	31	57.4
2	Morozova T.I. et al. [23]	2016	49	22	16	32.7	33	67.3
3	Balasanyantzh G.S. [24]	2017	14	26	2	14.3	12	85.7
4	Vasiljeva I.A. et al. [25]	2017	412	84	237	57.6	175	42.4
5	Lepshina S.M., Serduk O.V., Urovskaya E.I. [26]	2017	34	24	34	100	0	0
6	Konovalova N. M. et al. [27]	2017	21	80	10	41.6	11	52.3
7	Kondakova M.N. et al. [28]	2018	38	24	0	0	38	100
8	Tihonova L.U. et al. [29]	2018	23	24	13	56.5	10	43.5
9	Borisov S.E. et al. [30]	2019	315	24	315	100	n	n
10	Golubchikov P.N. et al. [31]	2019	39	48	8	20.5	31	79.5
11	Danilova T.I. et al. [32]	2020	46	24	16	34.8	30	65.2
12	Danilova T.I. et al. [32]	2020	34	24	10	29.4	24	70.6
13	Stavickaya N.V. et al. [33]	2020	70	70	70	100	0	0
14	Ivanova D.A. et al. [34]	2020	122	96	122	100	0	0
15	Yablonskiy P.K. et al. [35]	2022	23	96	0	0	23	100
16	Starshinova A.A. et al. [36]	2022	22	96	22	100	0	0
17	Morozova T.I. et al. [37]	2022	88	24	0	0	88	100
Total			1404		898	61.6	506	38.4

**Table 2:** General characteristics of treated patients with tuberculosis.

Patient Characteristics	Number of Patients with Symptoms/Total Number of Patients	%
**Pulmonary TB**		
Infiltrative TB	384/954	40.3
Fibrous-cavernous TB	295/954	30.9
Disseminated TB	80/954	8.3
Multiple tuberculomas	34/954	3.5
Caseous pneumonia	21/954	2.2
**Extrapulmonary TB**		
Intrathoracic lymphadenopathy	24/954	2.5
Generalized tuberculosis	14/954	1.4
Incident cases	266/909	29.3
Disease recurrence	213/909	23.4
Bacterial excretion	808/884	91.4
Concomitant pathology	703/851	82.6
HIV infection	42/703	5.9
**Addictions**		
Tobacco smoking	294/505	58.2
Alcohol addiction	139/694	20.1
Drug addiction	12/379	3.1

Note: tuberculosis (TB).

**Table 3:** Results of treatment of patients with tuberculosis with multiple- and extensive drug-resistance of the pathogen using bedaquiline.

Cure Rates	With MDR/XDR TB Patients	
	%	95% Cl
Clinical Efficacy	78.2	75.1–81.1
X-ray dynamic	72.6	67.3–77.5
Closure of cavities	34.9	28.1–42.2
Cessation of bacterial excretion	79.5	76.5–82.3
Treatment success	75.5	72.3–78.5
Recovery after a course of therapy	82.0	78.6–85.1
Departure from treatment	9.8	7.9–12.2
Death	6.5	4.9–8.3

**Table 4:** Comparison of the results of MDR and XDR TB treatment.

TB Patients	Group I—Patients with MDR Tuberculosis		Group II—Patients with XDR Tuberculosis	
	%	95% Cl	%	95% Cl
The efficiency of treatment by the end of the course of therapy	89.9	85.9–93.2	71.9	66.2–77.1
